# Perceived Stress Can Mediate the Associations between a Lifestyle Intervention and Fat and Fast Food Intakes

**DOI:** 10.3390/nu12123606

**Published:** 2020-11-24

**Authors:** Mei-Wei Chang, Roger Brown, Duane T. Wegener

**Affiliations:** 1 College of Nursing, The Ohio State University, Columbus, OH 43210, USA; 2 School of Nursing, University of Wisconsin-Madison, Madison, WI 53705, USA; brown3@wisc.edu; 3 Department Psychology, The Ohio State University, Columbus, OH 43210, USA; wegener.1@osu.edu

**Keywords:** stress, low-income women, fat intake, fast food intake, obesity

## Abstract

This secondary analysis study addressed a gap of knowledge: whether perceived stress reduction created by a lifestyle intervention might serve as a mediator for reducing fat and fast food intakes in low-income overweight or obese mothers of young children. This analysis included 338 low-income overweight or obese mothers of young children who completed a phone interview immediately after the 16-week lifestyle intervention. Valid surveys were used to assess perceived stress and fat and fast food intakes. Composite indicator structural equation modeling was performed to test the mediation effects. The overall effect of the intervention was not significant for fat intake but was significant for fast food intake (B = −0.53, *p* < 0.05). When assessing the potential role of perceived stress as a mediator, the indirect effects of the intervention on fat (B = −0.39, *p* < 0.01) and fast food (B = −0.27, *p* < 0.01) intakes were both significant. Future dietary intervention studies aimed to reduce fat and fast food intakes in low-income overweight or obese mothers of young children might consider including practical strategies aimed at reducing perceived stress.

## 1. Introduction

Nearly one in two American low-income women of child-bearing age are overweight or obese [1], especially those who give birth [2]. Being low-income increases risks for being overweight or obese [3], which is strongly associated with increased caloric intake (e.g., fast food intake) and cardiovascular disease [4]. Additionally, maternal obesity is associated with offspring health during childhood and later adult life [5,6]. Weight loss in overweight or obese women of child-bearing age is achievable via modification of dietary intake behaviors [7,8,9]. Most weight loss intervention studies have focused on decreasing dietary fat intake [10], an effective strategy for weight loss [11]. However, intervention engagement challenges low-income overweight or obese mothers of young children, partially due to high levels of perceived stress (hereafter, stress) [12,13].

There is compelling evidence that high levels of stress are associated with low-income, weight gain, and obesity [3,14,15,16]. Prior studies have consistently shown that living with chronic life stress, which is common in low-income individuals, is associated with greater preference for energy-dense foods, such as high-fat foods and fast foods [14,15,17,18]. Yet, it remains unknown whether intervention-based reduction in stress serves as a potential mediator of intervention effects on fat and fast food intake. Exploring whether stress can mediate intervention effect to reduce fat and fast food intakes in low-income overweight or obese mothers of young children is critically important to help researchers and clinicians provide appropriately targeted interventions, thus promoting maternal and child health outcomes.

Chang and colleagues conducted a randomized controlled community-based lifestyle behavior intervention (16-week) for low-income overweight or obese mothers of young children [19]. They found that the intervention significantly reduced stress [*p* < 0.01, Cohen D (*d)* = 0.34] [20], and fat (*p* < 0.05, *d* = 0.24) and fast food intakes (*p* < 0.05, *d* = 0.33) [21] at the end of the 16-week lifestyle intervention after adjusting baseline assessment. In the current secondary analysis, we conducted new analyses using cross-sectional data from 338 low-income overweight or obese mothers of young children (intervention group = 212, comparison group = 126) who completed the phone interview at the end of the 16 week-intervention. The objective of this paper is to examine whether stress serves as a potential mediator of the intervention effects on reducing fat and fast food intakes. We hypothesized that the intervention would have indirect effects on reducing fat and fast food intakes through reducing stress.

## 2. Materials and Methods

### 2.1. Study Design, Setting and Participants

A detailed description of the study procedure and study criteria has been previously reported [19,22]. Briefly, participants were recruited from the Special Supplemental Nutrition Program for Women, Infants, and Children (WIC) in Michigan between September 2012 and January 2015. WIC, a federally funded nutrition program in the US, provides food vouchers and nutrition education to low-income pregnant, postpartum, and breastfeeding women and children under 5 years old. The study recruiters personally invited women to be screened while waiting for their WIC appointments. Eligible women were Non-Hispanic White or African American, between 18 and 39 years old, between 6 weeks and 4.5 years postpartum, and have a body mass index (BMI) between 25.00–39.99 kg/m^2^ (using measured height and weight). They provided a written consent form before participating in the study. Michigan State University Institutional Review Boards approved the study procedure (10–970).

### 2.2. Intervention

Detailed descriptions of the intervention have been published previously [19]. The 16-week intervention aimed to prevent weight gain in low-income overweight or obese mothers of young children through the promotion of stress management, healthy eating, and physical activity. Intervention participants viewed a designated intervention video in DVD format at home (20 min/video, total of 10 videos: 4 stress management, 5 healthy eating, and 1 physical activity) and joined a peer support group teleconference (30 min/session, total of 10) every week for 4 weeks (Weeks 1–4) and then every other week (Weeks 6–16). To connect with the participants, the videos featured 4 overweight or obese WIC mothers of young children with their family members. They provided testimonies and demonstrated useful and practical tips to overcome daily challenges to better manage stress (e.g., taking deep breaths, counting to 10, and using positive talk), eat healthier (e.g., planning healthy meals and cooking healthy meals at home with a limited budget), and be more physically active (e.g., playing tag with young children outdoors and marching in place while watching TV). Peer educators and WIC dietitians who were trained in motivational interviewing led the peer support group teleconferences. The comparison group received reading materials consisting of general information about stress management, healthy eating, and physical activity.

### 2.3. Measures

Demographics were collected via a pencil-and-paper survey while women waited for their WIC appointments. Individual phone interviews were conducted to collect data on stress and fat and fast food intakes. There were no missing data in the present data set.

#### 2.3.1. Demographics

Women self-reported their own birthdate and that of their youngest child, both of which were used to calculate the study participants’ age and postpartum period, respectively. They also reported their race, education, employment and smoking status.

#### 2.3.2. Stress

Stress was measured using a validated Perceived Stress Scale (9 items) [23]. This survey measures the degree to which situations encountered during the past month in one’s life were appraised as stressful. Responses ranged from 1 (rarely or never) to 4 (usually or always). The overall stress score was the average of the 9 items with a higher score indicating a higher stress (mean = 2.48 (0.38), Cronbach alpha (α) = 0.73).

#### 2.3.3. Dietary Fat Intakes

Dietary fat intake was measured using a subscale of a valid Rapid Food Screener (17 items). The subscale was validated using a 100-item food frequency questionnaire with a general population and has moderate predictive validity assessed using Spearmen correlation (r = 0.69) [24]. Participants were asked about their fat intake (e.g., fried chicken, pizza, cookies, or ice cream) over the past 3 months. Responses were scored on a 5-point scale ranging from 0 (1 time per month or less) to 4 (5 or more times per week). The overall fat intake score was the sum of the 17 items with a higher score indicating higher frequency of high-fat food intake, hereafter, higher fat intake (mean = 18.35 (7.87); α = 0.77).

#### 2.3.4. Fast Food Intake

Fast food intake was measured using a subscale of a validated brief screener (12 items). The subscale was validated using three 24-h dietary recalls with adolescents aged 11–18 years old and has moderate predictive validity (r = 0.67) [25]. Participants were asked about how often they purchased foods at a restaurant (e.g., traditional fast food, Mexican fast food, or pizza restaurants and bakery/donut shop) where food was ordered at a counter or at a drive-through window. Responses ranged from 0 (never or rarely) to 8 (3 or more times/day). The overall fast food intake score was the sum of the 12 items with a higher score indicating higher frequency of fast food intake, hereafter higher fast food intake (mean = 19.79 (4.77); α = 0.66).

### 2.4. Statistical Analysis

**Figure 1:**
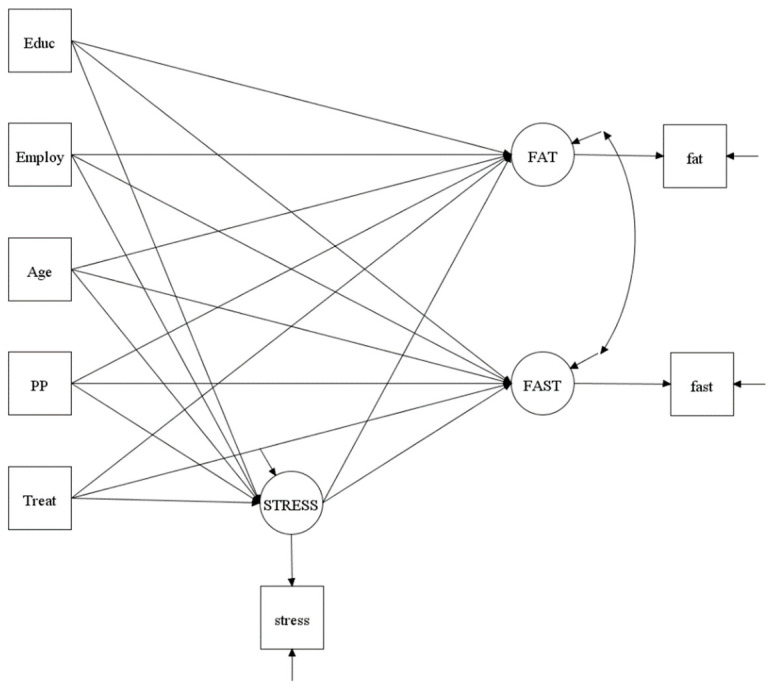
Structure of Mediation Model Testing. Squares are used to indicate manifest or single item variables, whereas circles are used to represent unmeasurable latent variables. Treat (intervention) was an exogenous variable (a predictor or an independent variable). FAT (fat intake) and FAST (fast food intake) were endogenous variables (outcome or dependent variables). STRESS (perceived stress) was a mediator (an endogenous variable). Educ (education), Employ (employment status), age, and PP (postpartum period) were covariates.

MPlus version 8.3 was used to conduct the statistical analyses [26]. T-test and Chi-square tests were performed to examine the group (intervention vs. comparison) differences in demographics. This secondary data analysis utilized data collected immediately after the 16-week intervention (cross-sectional data). Intervention was the exogenous variable (a predictor or an independent variable) and was dichotomized as 0 = comparison group and 1 = intervention group). Dietary fat and fast food intakes were the endogenous variables (outcome or dependent variables). Stress was the mediator (an endogenous variable in the analysis). Covariates included in the model testing were education, employment, age, and postpartum period because these demographics may affect the outcome measurements. A Composite Indicator Structural Equation (CISE) model using Bayesian estimation was used to test 2 mediational models: one for fat intake and one for fast food intake (Figure 1). Details regarding the Bayesian estimation approach in structural equation modeling have been described [27,28].

There are advantages of using CISE modeling because it improves reliability by incorporating measurement errors into the model, thereby reducing attenuation in estimates. The CISE modeling creates composite variables by combining the items of each separate measurement domain into a single indicator. To control for measurement errors in a CISE, the error variance of the indicator was fixed to (1 − α) * σ^2^, where α was Cronbach’s alpha, and σ^2^ was the variance of the composite variable. Additionally, aggregating items in CISE modeling promotes a normal distribution, reduces the number of measured variables in models by combining items for each variable, and improves the variable to sample size ratio.

Bayesian methods are a useful alternative to maximum likelihood estimation in mediation models, particularly with smaller samples [29]. We estimated the direct effects, indirect effects, and total effects using Empirical Bayesian (EB) Markov Chain Monte Carlo estimates, using data-derived estimates from our dataset as our EB informative priors [27,29,30]. Model fit were assessed using the Bayesian posterior predictive *p*-value (PPP) and prior PPP, where a value close to 0.5 indicates good fit [31], root mean square error of approximation (RMSEA, <0.05), comparative fit index (CFI, >0.90) and Tucker-Lewis index (TLI, >0.90). We utilized the proportion of maximum possible (POMP) scores in the endogenous variables with per unit change in the exogenous variables to compute effect size. POMP = [unstandardized parameter estimate/(maximum scale value − minimum scale value) + 1)] * 100 [32].

## 3. Results

### 3.1. Demographics

There were no significant differences between groups in mean BMI [intervention = 31.83 (4.34), comparison = 31.53 (4.28)], mean age [intervention: 29.21 (4.92), comparison: 29.63 (4.95)], race (white − intervention: 84.43%, comparison: 76.98%), education (at least some college education − intervention: 72.17%, comparison = 65.88%), and smoking (non-smokers–intervention: 82.55%, comparison: 77.78%). However, there were significant differences in postpartum periods [intervention: 1.63 y (1.23), comparison: 1.99 y (1.30, *p* < 0.05)] and employment status (employed full-or-part-time − intervention = 37.74%, comparison = 47.62%, *p* < 0.01).

### 3.2. Total Effects of Intervention and Covariates

**Figure 2:**
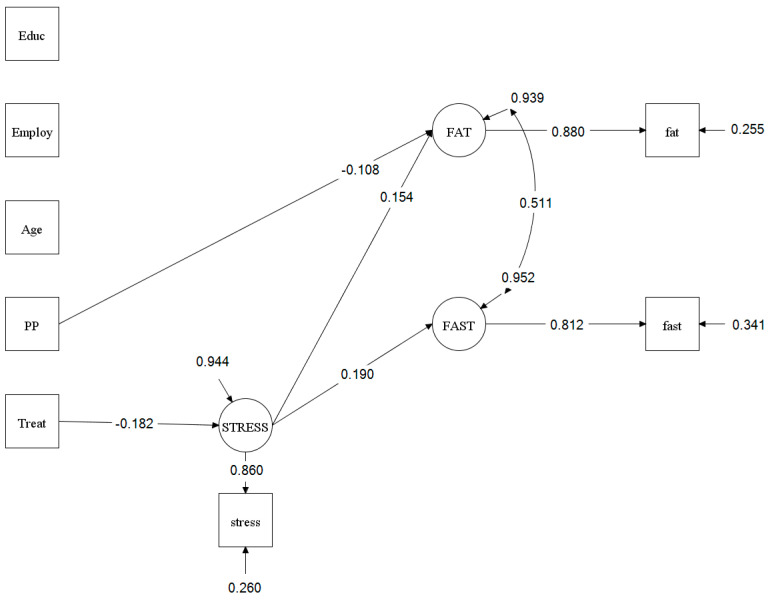
Significant Paths of Mediation Model Testing. Values presented in significant paths were standardized parameter estimate. Squares are used to indicate manifest or single item variables, whereas circles are used to represent unmeasurable latent variables. Treat (intervention) was an exogenous variable (a predictor or an independent variable). FAT (high fat intake) and FAST (fast food intake) were endogenous variables (outcome or dependent variables). STRESS (perceived stress) was a mediator (an endogenous variable). Educ (education), Employ (employment status), age, and PP (postpartum period) were covariates.

Without links between stress and fat or fast food intake in the model, the intervention did not reduce fat intake (top section of Table 1), which contradicts a published result using different analyses [21]. The non-significant finding is attributed to a negative indirect effect (intervention → stress → fat intake; Table 1 bottom section) added to a positive direct effect (intervention → fat intake; Table 1 middle section) resulting in a small and non-significant positive total effect (intervention → fat intake; Table 1 top section). Even without an overall effect on fat intake, it is still possible to have mediation (reported later) [33]. The intervention did significantly reduce fast food intake (B = −0.53, *p* < 0.05, POMP = −1.76%; see Table 1). Education, employment status, and age were not associated with stress or fat and fast food intakes. Postpartum period was not associated with stress or fast food intake but was significantly associated with fat intake (unstandardized parameter estimate (B) = 0.60, *p* < 0.05, see Figure 2). The PPP was 0.34 and prior PPP was 0.60, RMSEA = 0.04, CFI = 0.96, and TLI = 0.90, all of which indicate good model fit.

### 3.3. Direct and Indirect (Mediation) Effects

Figure 2 presents significant paths from the model testing, and Table 1 presents the result of mediation model testing. The intervention significantly reduced stress [B = −0.13, *p* < 0.01, POMP = −5.72%). When controlling for the intervention, higher stress was significantly associated with higher fat intake (B = 3.20, *p* < 0.001, POMP = 6.99%) and higher fast food intake (B = 2.19, *p* < 0.001, POMP = 7.28%). When controlling for stress, the intervention had no effects on fat or fast food intake. When assessing the potential role of stress as a mediator, indirect effects of the intervention on fat and fast food intakes through stress were significant and negative (fat intake: B = −0.39, *p* < 0.01, POMP = −0.84%; fast food intake: B = −0.27, *p* < 0.01, POMP = −0.88%).

## 4. Discussion

The present study is the first to examine whether stress might mediate the associations between a 16-week lifestyle intervention and fat and fast food intakes in low-income overweight or obese mothers of young children. We hypothesized that reducing stress would have indirect influences on reducing fat and fast food intakes through reductions in stress. The results of the present study supported the hypothesis: stress mediated the association between the intervention and fat intake and between the intervention and fast food intake.

Direct effects. When controlling for the intervention, the study findings suggest positive links between stress and fat and fast food intakes, which have been supported in isolation by 3 prior studies of low-income pregnant women [18,34], and low-income women of child-bearing age [15]. In the context of stress reduction helping to account for intervention effects on fat or fast food intake, the current findings might have important clinical implications. That is, they highlight the potential to intervene with high-stress women to promote stress management and thereby reduce fat and fast food intakes and improve maternal and child health outcomes. Using the link between stress and frequency of high fat intake (see Table 1, direct effects: stress → fat intake) as an example, a 1 point reduction in the stress scale (a 4- point scale) was associated with a nearly 7% (POMP) reduction in the frequency of high fat intake. If dietitians can help clients reduce stress by 2 points, their clients would, on average, report a 14% reduction in fat intake (i.e., 2 points × 6.99% lower fat intake/stress point reduction). To identify high-stress low-income overweight or obese mothers of young children, dietitians might consider using a visual analogue scale (asking clients to rate their stress on a single item with a 10-point scale), which is a quick and simple assessment of stress and eliminates potential problems with low literacy compared with using a survey-based assessment tool [35]. Then, dietitians can provide practical ways to help these women reduce stress at any time and location, e.g., taking deep breaths and counting to 10 before continuing a stressful activity. Additionally, dietitians might consider increasing clients’ coping self-efficacy to reduce stress [36] by promoting positive self-talk, e.g., “I have done this before; I can do it again”; “it is temporary; I can get through it.”

Mediation effects and direct effects. The current study results suggest that improvement in stress carried the effects of the intervention to fat and fast food intakes. The present findings also suggest a critical need to include stress management when a lifestyle intervention aims to reduce fat and fast food intakes in the target population. Direct comparison of our findings to prior studies is not feasible because this is the first study that included intervention as a predictor or independent variable to examine the mediation effects of stress on fat and fast food intakes. Future lifestyle intervention studies of the target population may consider examining such mediation effects.

## 5. Strengths

The present study included low-income overweight or obese mothers of young children, a group at high risk for obesity-related chronic conditions, who participated in a community-based lifestyle behavior intervention. The use of Bayesian methods to estimate the mediation effects further increases strength of this study.

## 6. Limitations

There are limitations to this study. This was a secondary data analysis undertaken after knowing that the intervention was effective in reducing stress, thereby making it important to replicate especially the stress mediation pattern in new research. Additionally, the analysis utilized cross-sectional data, thus, the causal-effect relationship cannot be assumed. Because of the nature of examining a measured mediator, we also cannot establish a causal link between stress and fat or fast food intake. Moreover, this study might be underpowered for the mediation analyses. Yet, no prior studies had evaluated whether stress mediated the associations between a lifestyle intervention and fat and fast food intakes. Therefore, the likely strength of such associations were difficult to anticipate (and the original study was not conducted with these mediational analyses in mind). However, the estimates from the current study could be used to design follow-up research powered to test a replication of the stress mediation. Additionally, brief dietary surveys were used to measure fat and fast food intakes instead of three 24-h dietary recalls or a long-form food frequency questionnaire. However, use of either measurement in this target population would create additional burden and likely reduce participation. Finally, results of this study may not be generalizable to low-income overweight or obese mothers in a different geographical location. Future lifestyle intervention studies of the target population should seek to replicate the current study findings with longitudinal data.

## 7. Conclusions

The current study extends the literature examining the direct association between stress and dietary intake. Stress apparently carried the effect of the lifestyle intervention to influence fat and fast food intakes in low-income overweight or obese mothers of young children. The results of the study highlight the imperative need to include stress management into lifestyle intervention studies aimed to reduce fat and fat food intakes for the target population.

## Figures and Tables

**Table 1:** Mediation model testing on 338 low-income overweight or obese mothers of young children (212 intervention, 126 comparison).

	B (SE)	95% CI	*p*	β	POMP
Total Effects of Intervention					
Intervention → Fat intake	0.05 (0.58)	−1.08, 1.18	0.93	0.01	0.10%
Intervention → Fast food intake	−0.53 (0.23)	−0.98, −0.10	0.016	−0.14	−1.76%
Direct Effects in Mediation Model					
Intervention → Stress	−0.13 (0.04)	−0.22, −0.04	0.004	−0.18	−5.72%
Stress → Fat intake	3.20 (0.96)	1.32, 5.10	<0.001	0.15	6.99%
Stress → Fast food intake	2.19 (0.65)	0.92, 3.45	<0.001	0.19	7.28%
Intervention → Fat intake	0.46 (0.55)	−0.60, 1.55	0.41	0.03	1.00%
Intervention → Fast food intake	−0.26 (0.13)	−0.63, 0.13	0.18	−0.03	−0.85%
Indirect Effects (Mediation)					
Intervention → Stress → Fat intake	−0.39 (0.19)	−0.84, −0.10	0.004	−0.06	−0.84%
Intervention → Stress → Fast food intake	−0.27 (0.13)	−0.57, −0.07	0.004	−0.07	−0.88%

*N* = 338. B = unstandardized parameter estimate, β = standardized parameter estimate. POMP = Proportion of maximum possible score in the endogenous variables per unit change in the exogenous variable. Treat (intervention) was an exogenous variable (a predictor or an independent variable). FAT (high fat intake) and FAST (fast food intake) were endogenous variables (outcome or dependent variables). STRESS (perceived stress) was a mediator (an endogenous variable). The analysis controlled for education, employment status, age, and postpartum status.
